# The relationship between *Plasmodium* infection, anaemia and nutritional status in asymptomatic children aged under five years living in stable transmission zones in Kinshasa, Democratic Republic of Congo

**DOI:** 10.1186/s12936-015-0595-5

**Published:** 2015-02-18

**Authors:** Vivi Maketa, Hypolite Muhindo Mavoko, Raquel Inocêncio da Luz, Josué Zanga, Joachim Lubiba, Albert Kalonji, Pascal Lutumba, Jean-Pierre Van geertruyden

**Affiliations:** Department of Tropical Medicine, University of Kinshasa, Kinshasa, Democratic Republic of Congo; International Health Unit, Faculty of Medicine, University of Antwerp, Antwerp, Belgium; Santé Rurale (SANRU), Kinshasa, Democratic Republic of Congo

**Keywords:** Plasmodium falciparum, Asymptomatic infection, Anaemia, Nutrition, Children under five, Democratic Republic of Congo

## Abstract

**Background:**

Malaria is preventable and treatable when recommended interventions are properly implemented. Thus, diagnosis and treatment focus on symptomatic individuals while asymptomatic *Plasmodium* infection (PI) plays a role in the sustainability of the transmission and may also have an impact on the morbidity of the disease in terms of anaemia, nutritional status and even cognitive development of children. The objective of this study was to assess PI prevalence and its relationship with known morbidity factors in a vulnerable but asymptomatic stratum of the population.

**Methods:**

A simple random sample, household survey in asymptomatic children under the age of five was conducted from April to September 2012 in two health areas of the health zone of Mont Ngafula 1, Kinshasa, Democratic Republic of Congo.

**Results:**

The PI prevalence were 30.9% (95% CI: 26.5-35.9) and 14.3% (95% CI: 10.5-18.1) in Cité Pumbu and Kindele health areas, respectively, (OR: 2.7; p <0.001). All were *Plasmodium falciparum* infected and 4% were co-infected with *Plasmodium malariae*. In Cité Pumbu and Kindele, the prevalence of anaemia (haemoglobin <11 g/dL) was 61.6% (95% CI: 56.6-66.5) and 39.3% (95% CI: 34.0-44.6), respectively, (OR: 2.5; p <0.001). The health area of Cité Pumbu had 32% (95% CI: 27.5-37.0) of chronic malnutrition (HAZ score ≤ −2SD) compared to 5.1% (95% CI: 2.8-7.6) in Kindele. PI was predictor factor for anaemia (aOR: 3.5, p =0.01) and within infected children, there was an inverse relationship between parasite density and haemoglobin level (β = −5*10^−5^, p <0.001). Age older than 12 months (aOR: 3.8, p = 0.01), presence of anaemia (aOR: 3.4, p =0.001), chronic malnutrition (aOR: 1.8, p = 0.01), having a single parent/guardian (aOR: 1.6, p =0.04), and the non-use of insecticide-treated nets (aOR: 1.7, p = 0.04) were all predictors for PI in the overall population.

**Conclusion:**

PI in asymptomatic children was correlated with anaemia and chronic malnutrition and was thus a harmful condition in the study population. Malaria control initiatives should not only focus on treatment of symptomatic infections but also take into consideration asymptomatic but infected children.

**Electronic supplementary material:**

The online version of this article (doi:10.1186/s12936-015-0595-5) contains supplementary material, which is available to authorized users.

## Background

Globally, according to the World Health Organization (WHO), malaria remains a major public health problem, affecting half the world’s population. There were an estimated 207 million episodes of malaria in 2012, of which approximately 81% occurred in the African region. The estimated number of deaths due to malaria was 627,000 in 2012, of which 90% were in Africa [1]. However, the real contribution may be higher due to the indirect morbidity rate through other infections, especially in vulnerable groups such as pregnant women and children under the age of five [2-7].

Malaria is preventable and treatable when recommended interventions are properly implemented. Effective preventive measures are: (i) intermittent preventive treatment for pregnant women (ii) indoor insecticide residual spraying and, (iii) the use of bed nets. These preventive measures may be more cost-efficient when combined with systematic screening and treatment of asymptomatic individuals in high-transmission settings [8]. In symptomatic individuals, early diagnosis based on microscopy or rapid diagnostic test and an efficacious treatment are necessary. In addition, all suspected cases of symptomatic *Plasmodium* infection (PI) should be biologically confirmed prior to treatment [9-14]. Thus, by definition, diagnosis and treatment are focused on symptomatic individuals while asymptomatic ones also play a role in the sustainability of the transmission, acting as a reservoir for the disease [15-19]. Furthermore, asymptomatic PI may have an impact on the morbidity of the disease in terms of anaemia, nutritional status and even cognitive development of children [2,4-7,20,21].

According to the health demographic survey carried out in the Democratic Republic of Congo (DRC) in 2007, almost two-thirds of anaemia cases and up to 85% of blood transfusions were likely due to episodes of SPI [22]. *Plasmodium* parasites mainly cause anaemia through hyperhaemolysis of red blood cells and the decrease in erythropoiesis operated through several pathways [23-31]. If the mechanisms of anaemia during PI are well documented, the relationship between PI and malnutrition still needs to be clarified [32]. Some authors suggest that malnutrition may lead to an exacerbation of morbidity or an increasing susceptibility to PI [33-36] while others report a protector effect of malnutrition on PI [37,38].

This paper aims to assess asymptomatic PI prevalence in the vulnerable group of children under the age of five living in malaria-endemic areas and to explore its relationship with morbidity factors such as anaemia and malnutrition status.

## Methods

### Study area

Kinshasa, capital of the DRC, has a surface of 13,195 sq km and more than eight million inhabitants [39]. The level of malaria transmission is high and perennial. The climate is tropical with two seasons, a rainy season of eight months and a dry season. Kinshasa is divided into six health districts: Nsele, Ndjili, Kalamu, Funa, Lukunga, and Gombe. Each district is further divided into health zones (HZs), the operational level of the health system in DRC, which are further divided into health areas (HAs). The total number of HZs in Kinshasa is 35 and the number of HAs is 384 [39]. This study was held to have complementary prevalence data on asymptomatic malaria as a Phase IIIb clinical trial was ongoing in the HZ of Mont Ngafula1 [40].

### Study design and study population

The household survey was conducted from April to September 2012 during the dry season. Two HAs, Kindele and Cité Pumbu, were randomly selected from the 16 HAs of Mont Ngafula1 using the RAND function of Microsoft Excel 2007 software. Random selection of households was based on a systematic sampling interval k, determined by dividing the number of households in the neighbourhood (obtained from the Civil Registry), by the number of neighbourhoods. The first household was randomly selected following the expanded programme on immunization (EPI) vaccine coverage survey method [41] and by considering the neighbourhood’s civil administration office as the central point of the neighbourhood. Asymptomatic children under the age of five were the targeted population. Questionnaires addressed to children’s the parents/guardians were used to collect information on demographic data and predictors of PI.

### Sample collection and laboratory analysis

To assess the prevalence of PI, blood for thick/thin smears and malaria rapid diagnostic test (RDT) were collected from the same finger prick. But this paper only report prevalence assessed by blood smears, results related to malaria RDT are reported elsewhere [42]. Blood smears were prepared on the same slide bearing a patient’s identification code. Slides were horizontally air-dried in a slide tray and stored in boxes. At the end of the day, blood slides were stained with 10% Giemsa, but the thin smears were previously fixed with pure methanol. Blood slides were read by experienced laboratory technicians at the Parasitology Laboratory, Kinshasa University. The parasite density (PD) was calculated by counting the number of asexual parasites per 200 leukocytes in the thick blood film. A laboratory technician counted 500 leukocytes before considering a slide as negative. When thick films were positive, thin films were read for species determination. Based on an assumed 8,000/μl white blood cells in children [43], the PD/μl was calculated using the following formula:

$$ Parasites/\mu l\ \left(P/\mu l\right) = \frac{\left( Number\  of\  parasites\  counted\ x\ 8,000\right)}{Number\  of\  leukocytes\  counted} $$

Haemoglobin concentration was measured in the laboratory using the Hemocontrol® device (EKF Diagnostics, Germany) with venous blood collected in the field and stored in vacutainer tubes containing EDTA. Anaemia was classified as mild, moderate and severe when haemoglobin concentrations were respectively 10.9-10.0 g/dl, 9.0-7.0 g/dl and <7 g/dl [44].

### Nutritional status

Height was measured to determine the height for age z-score (HAZ) according to the WHO growth standard references for children from zero to five years [45]. A HAZ score ≤ −2 standard deviation (SD) and ≤ −3 SD were defined as stunting or moderate chronic malnutrition and severe chronic malnutrition, respectively. The mid upper arm circumference (MUAC) was measured to assess the MUAC z-score according to the WHO growth standard references for children from zero to five years [46]. MUAC score ≤ −2 SD and ≤ −3 SD were defined as moderate and severe acute malnutrition respectively.

### Asymptomatic PI

Asymptomatic PI was assessed as the presence *P. falciparum* trophozoite on a blood smear and, the absence of temperature above 37.5 °C (axillary) or history of fever in the past 48 hours.

### Data analysis

Data were double-entered and validated in Epi info version 3.5.1. software and analysed using Stata version 11 (Stata Corp, College Station, Texas, USA). For the different statistical tests the level of significance was set at 5%. Progressive stepwise pr (0.10) backward pe (0.05) models were used to assess the predictor factors for the variables of interest.

### Ethical consideration

Ethical clearance was obtained by the Ethics Committee of the Public Health School of the University of Kinshasa. Written informed consent was collected from the parents or legal guardians prior to inclusion of the children. Thumb-printed consents were collected in the presence of an independent witness whenever the parents or legal guardians were illiterate. All children with a positive malaria RDT and/or blood smear were supplied with anti-malarial drugs as recommended by DRC national guidelines.

## Results

A total of 700 children under the age of five were included, 317 (45.3%) girls and 383 (54.7%) boys. The median age was 43.1 months (IQR: 28.0-54.6). The majority of the children (93.9%) were living in a household without screening on the windows. Moreover, 257 (36.7%) children were living in household that owned at least one insecticide-treated net (ITN) 162 (23.1%) slept under the ITN the night prior to data collection. Reasons given for the non-use of ITN by the parents were the heat (9.1%), children did not tolerate sleeping under the ITN (2.3%), the parents forgot to install the ITN (4.0%). but in the majority of the cases (84.6%), the parents gave no explanation. There was no significant difference regarding the proportion of ITN utilization within age group categories or HAs. Almost half of the children (49.4%) were living in a compound with more than eleven individuals and most of the households (55.9%) were composed of more than ten persons. The majority of parents were married or in couple (81.3%) with highest achieved educational level of secondary school (71.9%), and their professional occupations were diverse (Additional file 1).

The mean prevalence of PI in the two HAs was 23.1% (95% CI: 20.1-26.4), among them, 4.6% (95% CI: 3.0-6.1), 6.3% (95% CI: 4.4-8.0), 12.2% (95% CI: 9.8-14.7), respectively, had a PD <1,000/μl, PD ranging from 1,000/μl to 2,000/μl and PD >2,000/μl. The median PD was 2,357/μl (IQR: 1,120-6,868/μl, range: 32–137,600). Within the HAs, the prevalence of PI was 30.9% (95% CI: 26.5-35.9) and 14.3% (95% CI: 10.5-18.1), respectively, in Cité Pumbu and Kindele with OR 2.7 (p <0.001). In Cité Pumbu, the median PD was 2,000/μl (IQR: 1,121-6,400/μl; range: 32–137,600) while median PD was 3,360/μl (IQR: 1,120-10,849/μl; range: 48–49,098) in Kindele. There was a significantly lower proportion of children having PI in those sleeping under an ITN (p = 0.01) compared to those who did not slept under an ITN when the PD was above 2,000/μl (Table 1).

**Table 1:** **L Proportion of PI according to utilization of bed net in two health areas in Mont Ngafula1, Kinshasa, DRC, 2012**

**Sleep under ITN**	**Uninfected % (95% CI)**	**Overall PI % (95% CI)**	**PI <1000p/**μ**l % (95% CI)**	**PI 1000-2000p/**μ**l % (95% CI)**	**PI >2000p/**μ**l % (95% CI)**
***No n = 538***	74.5 (70.8-78.2)	25.5 (21.7-29.2)	4.3 (2.6-6.0)	6.9 (4.7-9.0)	14.3 (11.3-17.3)
***Yes n = 162***	84.5 (79.0-90.2)	15.5 (9.8-21.0)	5.6 (2.0-9.0)	4.3 (1.1-7.5)	5.6 (2.0-9.0)

Species identification revealed that *P. falciparum* was found in all positive smears, and mixed with *P. malariae* in 4% (29 slides) of cases. Among the samples, there were 1.6% (11 slides) which showed solely *P. falciparum* gametocytes, but they were considered a negative microscopic outcome.

The overall prevalence of anaemia in the two HAs was 51.1% (95% CI: 47.4-54.9) with 36.8% (95% CI: 33.3-40.4), 9.9% (95% CI: 7.6-12.1) and 4.4% (95% CI: 3.0-6.0) having, respectively, mild, moderate and severe anaemia. The median haemoglobin level in the study population was 10.9 g/dl (IQR: 9.8-11.9; range: 4.0-19.0). In Cité Pumbu and Kindele, the prevalence of anaemia was, respectively, 61.6% (95% CI: 56.6-66.5) and 39.3% (95% CI: 34.0-44.6), OR: 2.5, p <0.001. Median haemoglobin levels were 10.6 g/dl (IQR: 9.6-11.5, range: 4.0-19.0) and 11.5 g/dl (IQR: 10.4-12.2; range: 4.5-17.2), respectively, in Cité Pumbu and Kindele.

The prevalence of acute malnutrition was 14.7% (95% CI: 12.1-17.3) with 5.1% (95% CI: 3.5-6.8) and 9.6% (95% CI: 7.4-11.8) of children having, respectively, moderate and severe acute malnutrition. The HA of Kindele had a larger proportion of acute malnutrition (27.4%; 95% CI: 22.6-32.3) compared to Cité Pumbu (3.5%; 95% CI: 1.6-5.4) OR:10.4, p <0.001. The prevalence of chronic malnutrition was 19.6% (95% CI: 16.6-22.5) with 11.6% (95% CI: 9.2-13.9) and 1.0% (95% CI: 6.0-10.0) of children having, respectively, moderate and severe chronic malnutrition. The HA of Cité Pumbu had a larger proportion of chronic malnutrition of 32% (95% CI: 27.5-37.0) compared to 5.1% (95% CI: 2.8-7.6) in Kindele, OR: 8.7;p <0.0001.

### Predictors of PI

Age above 12 months, presence of anaemia, chronic malnutrition, having a single parent/guardian, head of household with a lower education, and the non-use of ITNs were predictor factors for PI in the overall population (Table 2). In Cité Pumbu the presence of anaemia, the presence of chronic malnutrition and a lower education of the household head were correlated with PI, while in Kindele, anaemia was the sole correlate (Table 2).

**Table 2:** **Predictor for PI (progressive stepwise (pr 0.10) backward pe (0.05) model) in asymptomatic children of two health areas of Mont Ngafula1, Kinshasa, DRC, 2012**

	**Population N = 700**		**Cité Pumbu n = 372**		**Kindele n = 328**	
**Variables**	**aOR**	**p value**	**aOR**	**p value**	**OR**	**p value**
Age (Months)						
≤12	1		1			
>12	3.8	0.01	5.1	0.003		
Anaemia						
No	1		1		1	
Yes	3.4	<0.001	3.4	<0.001	2.8	0.001
Chronic malnutrition						
No	1					
Yes	1.8	0.01				
Status of the parent/guardians						
In couple	1					
Single	1.6	0.04				
Education of the parents/guardians						
University			1			
Below university level			3.8	0.04		
Slept under ITN						
Yes	1		1			
No	1.7	0.04	2.2	0.01		

### Predictors of anaemia and nutritional status

**Figure 1:**
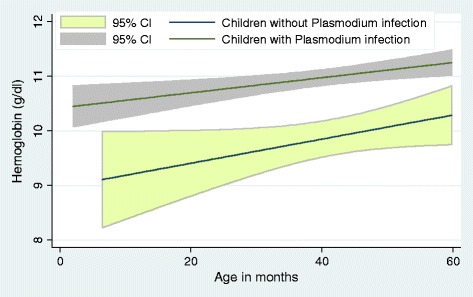
**Fitted line plot of haemoglobin concentration (g/dl) in children (age/month) with or without asymptomatic parasitaemia in two health areas of Mont Ngafula 1, Kinshasa, DR Congo, 2012.**

**Figure 2:**
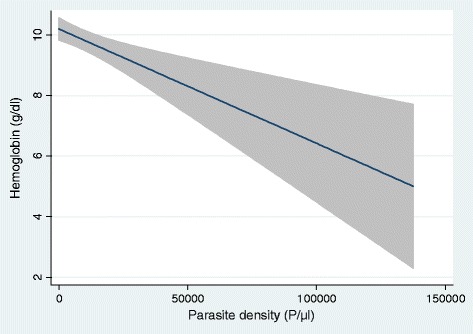
**Fitted line plot of haemoglobin concentration (g/dl) according to the parasite density (p/**μ**l) in children under the age of five in two health areas of Mont Ngafula 1, Kinshasa, DR Congo, 2012 (shaded area is 95%CI).**

In the overall population, children older than 12 months, having PI and having a non-employed household head were significantly more at risk of anaemia (Table 3). Figure 1 shows that uninfected children have a significantly higher haemoglobin level in general, regardless of age, indicating that children with parasitaeamia had higher risk of anaemia (OR: 3.6, p =0.001). Moreover, within the infected children group, an inverse relationship between PD and haemoglobin level was observed (β = −5*10^−5^, p <0.001) (Figure 2).

**Table 3:** **Predictor for anaemia (progressive stepwise (pr 0.10) backward pe (0.05) model) in two health areas of Mont Ngafula1, Kinshasa, DRC, 2012**

	**Population N = 700**		**Cité Pumbu n = 372**		**Kindele n = 328**	
**Variables**	**aOR**	**p value**	**aOR**	**p value**	**aOR**	**p value**
Age (months)						
>12	1		1			
≤12	2.6	0.01	2.8	0.01		
PI						
No	1		1		1	
Yes	3.5	0.001	3.6	<0.001	2.9	0.001
Profession of the household head						
Salaried employee	1		1			
Other	2.0	0.001	2.2	0.078

In the study population, the absence of screening on the windows was shown as a predictor for acute malnutrition while presence of PI and having a non-employed household head were predictors for chronic malnutrition (Table 4).

**Table 4:** **Predictor for acute and chronic malnutrition (progressive stepwise (pr 0.10) backward pe (0.05) model) in health zone of Mont Ngafula1, Kinshasa, DRC, 2012**

**Variables**	**Acute malnutrition (MUAC)**		**Chronic malnutrition (HAZ-score)**	
	**aOR**	**p value**	**aOR**	**p value**
PI				
No	--	--	1	
Yes	--	--	1.9	0.004
Profession of the household head				
Salaried employee	--	--	1	
Other	--	--	2.2	0.02
Presence of screening on windows				
Yes	1		--	--
No	2.4	0.03	--	--

## Discussion

The prevalence of PI in the two HAs of the HZ of Mont Ngafula I was different between Cité Pumbu and Kindele. Children in Cité Pumbu were almost three times more at risk of having parasitaemia. Therefore the results may not be representative for the whole HZ because of the high heterogeneity within the HAs. This difference may be explained by microclimatic differences between the two HAs, such as transmission intensity, as in endemic contexts the proportion of children with PI has spatio-temporal variances [47,48]. There were 1.6% of children who carried only gametocytes, this condition can be due to anti-malarial treatment prior to the visit [49-51] although no parent/guardian mentioned it. In the field, report of previous medication by parents is not always effective. For example, a study held in Tanzania detected anti-malarial drugs in the blood of 80% of screened children under the age of five, despite the fact no parents reported an intake of anti-malarials in the previous 28 days [52]. In the context of this study, as it was stated in the consent form that children with PI would be treated despite the absence of fever, one might assume that parents/guardians preferred to hide information about previous drug intake to ensure their children would benefit from the study medication, as noticed on the clinical trial site (Kalabuanga *et al*. personal communication).

Children under one year of age were protected from asymptomatic infection. One might assume an intensive use of bed nets in that age group but there was no difference according to the use of ITNs within age groups. In younger children, especially under six months old, the presence of maternal antibodies may decrease the prevalence of PI and therefore of asymptomatic infection [53,54], while in older infants, over six months, when maternal protection wanes, asymptomatic infection may be uncommon as the lack of acquired anti-malaria immunity deviates these children immediately to symptomatic or even severe symptomatic malaria episodes, decreasing the prevalence of asymptomatic infection in these infants [55,56].

The use of ITNs reduces the contact between host and vector and as shown in this study, children sleeping under ITNs were significantly less at risk of PI. There is evidence in the literature of the positive impact of their use on reduction of disease [15,57-59], but, paradoxically, the protection of ITNs brings the fear of shifting vulnerability to malaria onto school-aged children: on the one hand, schoolchildren may use ITNs less, and on the other hand, an impaired acquisition of immunity to malaria due to diminished contact with *Plasmodium* at younger age [58,60-64]. ITNs were efficient in preventing parasitaemia only when PD was ≥2,000/μl, which might explain the decrease of mortality due to malaria by the use of ITNs [65], as patients with symptomatic PI tend to have a higher PD than asymptomatic subjects [66]. However, the finding that ITNs did not affect the overall proportion of asymptomatic children with PI is intriguing. A low PD might be considered a benefit since it could act, similarly to the use of intermittent preventive treatment, as a blood stage vaccine [67,68]. Unfortunately, it can be argued that PI alone, whatever the PD is, is a harmful condition because of its impact at community level on the sustainability of transmission [2,8]. Moreover, a longitudinal study performed in Uganda showed that asymptomatic children with PI were almost five times more at risk to develop a clinical episode of malaria within 30 days than children without parasitaemia [69]. In the study population, children with PI had significantly lower haemoglobin levels. Analysis revealed that PD and haemoglobin levels were inversely correlated with children more likely to suffer from severe anaemia when they are highly infected. However, in a tropical context, with interaction between many factors, anaemia cannot be said to be a consequence of PI alone [70] but, in endemic settings, an assumption could be that anaemia might be related to former PI episodes. In face of such evidence it is clear that asymptomatic PI may be a harmful condition due to its direct and public health impact on haemoglobin levels, particularly in the vulnerable group of children under five [71-74]. In the study population, chronic malnutrition and anaemia were all correlated, nevertheless, the causality chain, cannot be determined through a cross-sectional design. Longitudinal studies are needed to answer this question. A limitation of this study is a possible underestimation of the prevalence of PI because of its assessment with a BS instead of PCR [75].

The prevalence of acute and chronic malnutrition were 15 and 20%, respectively, which is higher than the prevalence previously reported in Kinshasa (4%) in the same age group [22], but this may be due to ecological effect as this study included only two HAs of a city that has 384 HAs in total. As in an endemic setting, asymptomatic PI although can be considered an intermittent chronic condition; longitudinal studies are also needed to assess its the relation with nutritional status. The high heterogeneity within the two HAs in terms of nutritional status may be related to different socio-economical patterns in the two HAs, but, again, other studies are needed to explore these issues.

## Conclusion

PI although asymptomatic was correlated with anaemia and chronic malnutrition and is thus a harmful condition in the children under the age of five years. Thus, malaria control initiatives should also target asymptomatic PI to reduce malaria-associated morbidity.

## Additional file

Additional file 1: Table S1. Demographic characteristic and median parasite density of Plasmodium falciparum of asymptomatic children in 2 health areas of Mont Ngafula1, Kinshasa, DRC, 2012.

## Abbreviations

PI: *Plasmodium* infection
CI: Confidence interval
DRC: Democratic Republic of Congo
EPI: Expanded programme on immunization
HA: Health area
HAZ: Height for age z-score
HZ: Health zone
IQR: Interquartile range
ITN: Insecticide-treated net
MUAC: Mid upper arm circumference
PD: Parasite density
RDT: Rapid diagnostic test
SPI: Symptomatic *Plasmodium* infection
