# Supplementary material for: The relationship between Plasmodium infection, anaemia and nutritional status in asymptomatic children aged under five years living in stable transmission zones in Kinshasa, Democratic Republic of Congo
Source: Malar J. 2015 Feb 18;14:83. doi: 10.1186/s12936-015-0595-5 (PMC4336722; doi:10.1186/s12936-015-0595-5)
Supplement: Additional file 1: Table S1. — Demographic characteristic and median parasite density of Plasmodium falciparum of asymptomatic children in 2 health areas of Mont Ngafula1, Kinshasa, DRC, 2012. [file 12936_2015_595_MOESM1_ESM.docx]

**Additional file 1: Table S1: Demographic characteristic and median parasite density of *Plasmodium* *falciparum* of asymptomatic children in 2 health areas of Mont Ngafula1, Kinshasa, DRC, 2012**

|  | **Cité Pumbu** | | | **Kindele** | | |
| --- | --- | --- | --- | --- | --- | --- |
| ***Variables*** | ***n=372*** | **API** | **PD (p/µl)** | ***n=328*** | **API** | **PD (p/µl)** |
|  | n (%) | n (%) | M (IQR) | n (%) | n (%) | M (IQR) |
| ***Gender*** |  |  |  |  |  |  |
| ***Female*** | 152 (40.9) | 47 (30.9) | 2,456 (1,249-6,464) | 165 (50.3) | 23 (13.9) | 3,360 (1,113-6,868) |
| ***Male*** | 220 (59.1) | 68 (30.9) | 1,905 (1,073-5,957) | 163 (49.7) | 24 (14.7) | 3,640 (1,132-12,584) |
| ***Age (months)*** |  |  |  |  |  |  |
| ***≤12*** | 35 (9.4) | 4 (11.4) | 14,521 (480-40,201) | 6 (1.8) | 0 (0.0) | --- |
| ***13-24*** | 52 (14.0) | 10 (19.2) | 2,729 (1,970-15,320) | 27 (8.2) | 6 (22.2) | 3,400 (560-9,965) |
| ***25-36*** | 69 (18.5) | 21 (30.4) | 2,361 (1,600-5,360) | 82 (25.0 | 18 (22.0) | 2,363 (1,120-4,350) |
| ***37-48*** | 72(19.4) | 23 (31.9) | 1,984 (1,294-4,320) | 69 (21.1) | 9 (13.0) | 4,139 (1,648-15,781) |
| ***49-60*** | 144 (38.7) | 57 (39.6) | 1,726 (930-5,514) | 144 (43.9) | 14(9.7) | 5,454 (1,556-12,088) |
| ***Anaemia*** |  |  |  |  |  |  |
| ***Absent*** | 143 (38.4) | 24 (16.8) | 1,184 (640-3,077) | 199 (60.7) | 18 (9.1) | 4,244 (1,200-12,088) |
| ***Mild*** | 170 (45.7) | 59 (34.7) | 1,916 (1,255-6,480) | 88 (26.8) | 19 (21.6) | 1,144 (704-4,268) |
| ***Moderate*** | 42 (11.3) | 20 (47.6) | 3,554 (1,988-9,195) | 27 (8.2) | 8 (29.6) | 8,559 (2,803-20,697) |
| ***Severe*** | 17 (4.6) | 12 (70.6) | 9,374 (1,434-22,171) | 14 (4.3) | 2 (14.3) | 24,260 (9,965-38,554) |
| ***Acute malnutrition*** |  |  |  |  |  |  |
| ***Absent*** | 359 (96.5) | 110 (95.7) | 1,995 (1,109-5,360) | 238 (72.6) | 33 (70.2) | 4,000 (1,200-10,849) |
| ***Moderate*** | 9 (2.4) | 4 (3.5) | 16,160 (1,722-38,434) | 27 (8.2) | 4 (8.5) | 1,064 (904-1,760) |
| ***Severe*** | 4 (1.1) | 1 (0.9) | 7,680 (7,680-7,680) | 63 (19.2) | 10 (21.3) | 4,715 (560-15,780) |
| ***Chronic malnutrition*** |  |  |  |  |  |  |
| ***Absence*** | 252 (67.7) | 69 (60.0) | 2,400 (1,037-6,464) | 311 (94.8) | 45 (95.7) | 3,360 (1,120-9,965) |
| ***Mild*** | 67 (18.0) | 25 (21.7) | 1,916 (1,440-4,000) | 14 (4.3) | 2 (4.3) | 6,548 (1,008-12,088) |
| ***Severe*** | 53 (14.3) | 21 (18.3) | 1,726 (1,255-8,709) | 3 (0.9) | 0 (0.0) | --- |
| ***Individuals per household*** |  |  |  |  |  |  |
| ***≤5*** | 108 (29.1) | 29 (26.6) | 2,361 (1,142-6,400) | 88 (26.8) | 12 (13.6) | 11,469 (2,408-20,697) |
| ***6-10*** | 73 (19.6) | 24 (32.9) | 2,692 (1,331-5,397) | 40 (12.2) | 9 (22.5) | 1,648 (1,067-4,528) |
| ***≥11*** | 191 (51.3) | 62 (30.9) | 1,800 (1,121-7,680) | 200 (61.0) | 26 (13.0) | 2,843 (1,113-5,600) |
| ***Status of the parent/guardians*** |  |  |  |  |  |  |
| ***In couple*** | 281 (75.5) | 81 (28.8) | 2,195 (1,280-10,510) | 288 (87.8) | 39 (13.5) | 9,748 (1,552-31,329) |
| ***Single*** | 91 (24.5) | 34 (37.4) | 2,000 (960-5,515) | 40 (12.2) | 8 (20.0) | 3,280 (1,120-6,868) |
| ***Education of the parent/guardians*** |  |  |  |  |  |  |
| ***Primary school*** | 41 (11.0) | 16 (39.0) | 2,409 (1,705-18,150) | 44 (13.4) | 6 (13.6) | 1,520 (800-3,280) |
| ***Secondary*** | 304 (81.7) | 96 (31.6 | 1,986 (1,115-5,437) | 199 (60.7) | 30 (15.1) | 2,880 (1,113-1,5781) |
| ***University*** | 27 (7.3) | 3 (11.1) | 1,991 (512-3,960) | 85 (25.9) | 11 (12.9) | 4,528 (2,416-9,965) |
| ***Profession of the parent/guardians*** |  |  |  |  |  |  |
| ***Unemployed*** | 131 (35.2) | 32 (24.4) | 2,181 (1,179-4,109) | 45 (13.7) | 6 (13.3) | 1,602 (1,200-4,139) |
| ***Independent*** | 107 (28.8) | 41 (38.3) | 2,006 (1,014-8,709) | 102 (31.1) | 14 (13.7) | 4,195 (1,840-12,088) |
| ***Salaried employee*** | 25 (6.7) | 8 (32.0) | 1,619 (570-2,697) | 93 (28.4) | 12 (12.9) | 4,134 (1,697-10,407) |
| ***Other*** | 109 (29.3) | 34 (312) | 2,185 (1,280-6,480) | 88 (26.8) | 15 (17.1) | 1,200 (800-22,693) |
| ***Screen on windows*** |  |  |  |  |  |  |
| ***No*** | 348 (93.5) | 110 (31.6) | 2,003 (1,121-6,400) | 309 (94.2) | 47 (15.2) | 3,360 (1,120-10,849) |
| ***Yes*** | 24 (6.5) | 5 (20.8) | 1,991 (1,840-2,456) | 19 (5.8) | 0 (0.0) | --- |
| ***ITN ownership*** |  |  |  |  |  |  |
| ***No*** | 239 (64.3) | 84 (35.2) | 1,997 (1,248-5,880) | 204 (62.2) | 27 (13.2) | 4,139 (2,327-10,849) |
| ***Yes and slept under*** | 101 (27.1) | 18 (17.8) | 1,722 (640-5,515) | 61 (18.6) | 7 (11.5) | 1,200 (1,008-5,600) |
| ***Yes but did not slept under*** | 32 (8.6) | 13 (40.6) | 4,030 (1,440-6,464) | 63 (19.2) | 13 (20.6) | 1,648 (800-18,700) |
